# Acute Dental Periapical Abscess and New-Onset Atrial Fibrillation: A Nationwide, Population-Based Cohort Study

**DOI:** 10.3390/jcm10132927

**Published:** 2021-06-30

**Authors:** Amaar Obaid Hassan, Gregory Y. H. Lip, Arnaud Bisson, Julien Herbert, Alexandre Bodin, Laurent Fauchier, Rebecca V. Harris

**Affiliations:** 1 Department of Public Health, Policy and Systems, Institute of Population Health, University of Liverpool, Liverpool L69 3GL, UK; amaar.hassan@liverpool.ac.uk (A.O.H.); harrisrv@liverpool.ac.uk (R.V.H.); 2 Liverpool Centre for Cardiovascular Science, University of Liverpool and Liverpool Heart & Chest Hospital, Liverpool L69 7TX, UK; 3 Aalborg Thrombosis Research Unit, Department of Clinical Medicine, Aalborg University, 9000 Aalborg, Denmark; 4 Service de Cardiologie, Centre Hospitalier Universitaire Trousseau Faculté de Médecine, Université François Rabelais, 37044 Tours, France; arnaud.bisson37@gmail.com (A.B.); j.herbert@chu-tours.fr (J.H.); alexandrebodin.mail@gmail.com (A.B.); laurent.fauchier@univ-tours.fr (L.F.)

**Keywords:** atrial fibrillation, periapical abscess, oral health, acute dental infection, cardiovascular disease

## Abstract

There are limited data on the relationship of acute dental infections with hospitalisation and new-onset atrial fibrillation (AF). This study aimed to assess the relationship between acute periapical abscess and incident AF. This was a retrospective cohort study from a French national database of patients hospitalized in 2013 (3.4 million patients) with at least five years of follow up. In total, 3,056,291 adults (55.1% female) required hospital admission in French hospitals in 2013 while not having a history of AF. Of 4693 patients classified as having dental periapical abscess, 435 (9.27%) developed AF, compared to 326,241 (10.69%) without dental periapical abscess that developed AF over a mean follow-up of 4.8 ± 1.7 years. Multivariable analysis indicated that dental periapical abscess acted as an independent predictor for new onset AF (*p* < 0.01). The CHA_2_DS_2_VASc score in patients with acute dental periapical abscess had moderate predictive value for development of AF, with Area Under the Curve (AUC) 0.73 (95% CI, 0.71–0.76). An increased risk of new onset AF was identified for individuals hospitalized with dental periapical abscess. Careful follow up of patients with severe, acute dental periapical infections is needed for incident AF, as well as investigations of possible mechanisms linking these conditions.

## 1. Introduction

Atrial Fibrillation (AF) is the commonest cardiac rhythm disorder (affecting about 1–3% of the population) [1,2]. Sufferers are 3–5 times more times likely to develop stroke, heart failure and myocardial infarction [3]. Although people with AF can suffer a variety of symptoms such as chest pain, dizziness and fatigue, many patients are asymptomatic and only present late with advanced and serious heart problems or a stroke [4]. Population screening programmes are therefore important to pick up cases early. Although AF represents a significant cause of population mortality and morbidity, as well as health care expenditure, the latter is set to escalate due to an ageing population profile given that AF is more prevalent in older people [4]. There are many risk factors for AF, such as: ageing, sex (male), diabetes mellitus, and hypertension [5]. However, many of the risk factors associated with AF are also linked to poor oral health. Indeed, the latter may be a potential causative factor for AF since inflammatory markers generated by oral diseases are found to have a direct effect on the cellular function and electrophysiological remodelling of the heart [6,7].

The main oral disease which has so far been studied in relation to CVD is periodontitis, which is defined as inflammation of the gums and destruction of the surrounding bone. Most adults have some form of gum disease and 11.2% have suffer with severe periodontitis [8]. Severe periodontal disease and tooth loss together affects around 3.9 billion people globally and is estimated as the 6th most frequent global disease [9,10]. Studies show biomarkers such as IL-6 and CRP, which are implicated in both cardiovascular disease (CVD) and periodontitis, are linked to AF [11,12]. Other studies also report a reduction in inflammation following periodontal treatment [13,14], giving rise to a consensus that there is moderate evidence of a decrease in inflammatory biomarkers following treatment of periodontitis [15].

More recently, oral diseases related to CVD apart from periodontitis have also been investigated, such as apical (or periapical) periodontitis, which is an infection of the tooth and root canal system leading to destruction of the surrounding tissues [16]. More than half the population have been found to have apical periodontitis on at least one tooth, which is often presented as a chronic disease [17]. A systematic review reported an association between CVD and chronic apical infection, which suggests that while oral infections elicit a local tissue response, there are wider, systemic effects [18]. However, the primary studies included in the review only studied asymptomatic and chronic apical infections diagnosed through radiographic imaging, and AF was not included as separate entity within the study of CVD effects. Studies investigating oral diseases with CVD do not generally include AF as an outcome for analysis, although one recent retrospective study found that patients with a history of AF had a statistically significant incidence of diagnosed apical infection [19]. Of note, there is a positive association for reduced endothelial function with chronic apical infections [19,20] (and participants were also free of periodontitis), and given the associations between AF and endothelial dysfunction, this may have implications for increasing AF incidence [21].

There are a few retrospective cohort studies linking oral diseases with AF [22,23,24]. A recent 17-year longitudinal study has also found severe periodontitis with higher associations with AF (multivariable adjusted HR, 1.31, 95% CI, 1.06–1.62) [25]. One case report described a patient admitted with acute dental abscess and urticaria, that had also developed persistent AF, although the AF diagnosis could have been a coincidence [26].

Thus far, studies have only focused on asymptomatic, chronic low-grade inflammatory processes associated with oral disease, such as chronic periodontitis, rather than symptomatic or severe dental infections [23], although a recent systematic review [27] concluded there is a need for further research in this area. Incidence of acute dental infections and hospital attendance are common; 5–46% of all people suffer with an acute periapical abscess in their lifetime and 0.7% of all emergency attendances are related to dental problems [28,29,30]. Acute periapical abscess is more prevalent for people in disadvantaged groups and can have a significant impact causing sleepless nights, missed work and reduced quality of life [31].

In the present study, we aimed to investigate the relationship of acute dental periapical abscess with new-onset AF in a nationwide cohort study. We tested the hypothesis that acute periapical abscess is associated with incident AF, in relation to risk strata (using the CHA_2_DS_2_VASc score) using data from the French national hospital database from the Program de Médicalisation des Systèmes d’Information (PMSI) [32].

## 2. Materials and Methods

### 2.1. Study Design

This was a retrospective longitudinal cohort study which covers the full population and admissions across 1546 hospitals in France. All patients in France are discharged using the PMSI database and categorised with codes relating to diagnosis and treatment, as in the US Medicare system [33]. Patients are linked with unique identifiers for multiple admissions, ensuring the data remains anonymised. The inpatient International Classification of Diseases (ICD) codes were captured and commonly used for gathering large amounts of data [34].

The population for analysis was taken from French hospitals using the PMSI database, which included adults who were admitted from 01 January until 31 December 2013 and had at least five years of follow up, unless deceased. The Tenth Revision codes, International Classification of Diseases (ICD-10 codes) were used to identify primary and secondary diagnosis specified as infection inside and surrounding the root of the tooth and including hospital admission (dental periapical abscess K04.6, K04.7) [35]. After exclusion of patients with a history of previous AF, patients were followed up for new-onset AF with hospitalisation using ICD-10 codes. Identifying AF using an electronic database is considered reliable, and PMSI data have been evaluated in previous studies [32,36]. Baseline characteristics from patients hospitalised during 2013 with or without dental periapical abscesses were recorded, in addition to age, gender, and medical history. Events and predictors for AF were monitored with at least 5 years follow up. We also calculated the CHA_2_DS_2_VASc score (congestive heart failure, high blood pressure, diabetes, stroke, heart disease, age, gender) which is used for stroke risk stratification, but used this to stratify risk for new-onset AF where acute periapical abscess was present [34,35,36,37].

### 2.2. Ethical Approval

The research database involved a collection of non-identifiable information used for data analysis with external researchers. The study was conducted retrospectively and, as patients were not involved in its conduct, there was no impact on their care. Studies such as this study have been labelled as clinical audits, therefore sponsorship was not required from the University of Liverpool or the University of Tours [32,37,38]. Comparable to previous articles, informed consent was not obtained as the data were retrospective [22,23]. All patient information remained confidential and anonymised. The French law allows approval of certain public institutions, such as large university hospitals, to self-declare authorisation and access to confidential information when using electronic data for research purposes. The French Data Protection Authority have granted access to the PMSI data between 2008 and 2015, for the purpose of assessing the prevalence and incidence of cardiovascular diseases and their complications to the University of Tours in France. Procedures for data collection and management were approved by the Commission Nationale de l’Informatique et des Libertés (CNIL), the independent National Ethical Committee protecting human rights in France, which ensures that all information is kept confidential and anonymous (authorisation number 1897139).

### 2.3. Statistical Analysis

Baseline characteristics of patients were given as patient number (N) and percentages (%), or mean ± standard deviation, and included CHA_2_DS_2_VASc scores with average year follow ups using mean, median and interquartile range. Predictors for new-onset AF were calculated during follow up for the whole population and for patients with dental periapical abscess, which were assessed using univariate and multivariable Cox regression models. For patients with no previous dental periapical abscess, data were censored in case of dental periapical abscess during follow-up at the date of this event. Incidence and hazard ratio predictions, with confidence interval (95% CI) and *p* value (<0.05) for new-onset atrial fibrillation were calculated in patients with dental periapical abscess during follow up, using different CHA_2_DS_2_VASc scores. The specificity and sensitivity for CHA_2_DS_2_VASc scores as a predictor for new-onset AF and dental periapical abscess were examined further by plotting the Area Under the Curve-Receiver Operating Characteristic (AUC-ROC). All comparisons with *p* < 0.05 were considered statistically significant. All analyses were performed using Enterprise Guide 7.1, (SAS Institute Inc., SAS Campus Drive, Cary, NC, USA), USA and STATA version 12.0 (Stata Corp, College Station, TX, USA).

## 3. Results

**Figure 1:**
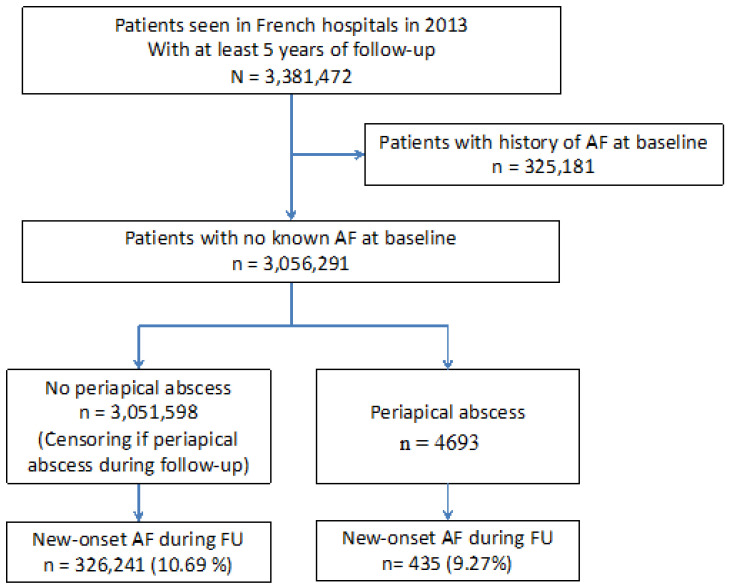
Flow chart of the study patients.

In total, 3,381,472 patients were identified in French hospitals during 2013 that attended emergency services and required hospital admission. The study population included at least five years of follow-up, unless deceased, and excluded patients with history of previous AF. A flowchart of study enrolment is shown in Figure 1. Of patients at baseline, 4693 were identified with a history of dental periapical abscess requiring hospitalisation. During a mean follow up period of 4.8 ± 1.7 years (median 5.5, IQR 5.1–5.8 years), there were 435 (9.27%) patients with a history of dental periapical abscess that were diagnosed with new-onset AF, compared to 326,241 (10.69%) without dental periapical abscess that developed AF.

Baseline characteristics of patients are shown in Table 1. Patients with a history of a dental periapical abscess were older (*p* value < 0.0001) and were more often male (*p* value < 0.0001). The prevalence of comorbidities for patients with dental periapical abscess was higher, including hypertension, diabetes mellitus, heart failure, obesity, alcohol related diagnosis, chronic kidney disease, and lung disease (*p* value < 0.0001, respectively). As shown in Table 2, CHA_2_DS_2_VASc scores were higher for those with periapical abscess at baseline (*p* value < 0.01).

The multivariable analysis represented in Table 3 found periapical abscess to be an independent predictor for new-onset AF, hazard ratio (HR) 1.11 (95% confidence interval (CI), 1.01–1.22). Other significant predictors for new-onset AF included, among others, older age, male sex, hypertension, diabetes mellitus, heart failure, aortic stenosis, obesity, and smoking (*p* value < 0.0001, respectively). For patients with a dental periapical abscess, there were positive associations for new-onset AF with older age, male sex, hypertension, aortic regurgitation, dilated cardiomyopathy, previous pacemaker or defibrillator and inflammatory disease (all *p* value < 0.05) using multivariable analysis, see Table 4.

The incidence (per 100 person-years) of new-onset AF generally increased with higher CHA_2_DS_2_VASc scores for patients with a history of dental periapical abscess, see Table 5. The HRs for new-onset AF in patients with dental periapical abscess using different CHA_2_DS_2_VASc scores (in comparison to patients with a score of zero) are shown in Table 6. As expected, patients with a CHA_2_DS_2_VASc score 8 had the highest HR of 34.2.0 (95% CI, 8.3–140.8).

**Figure 2:**
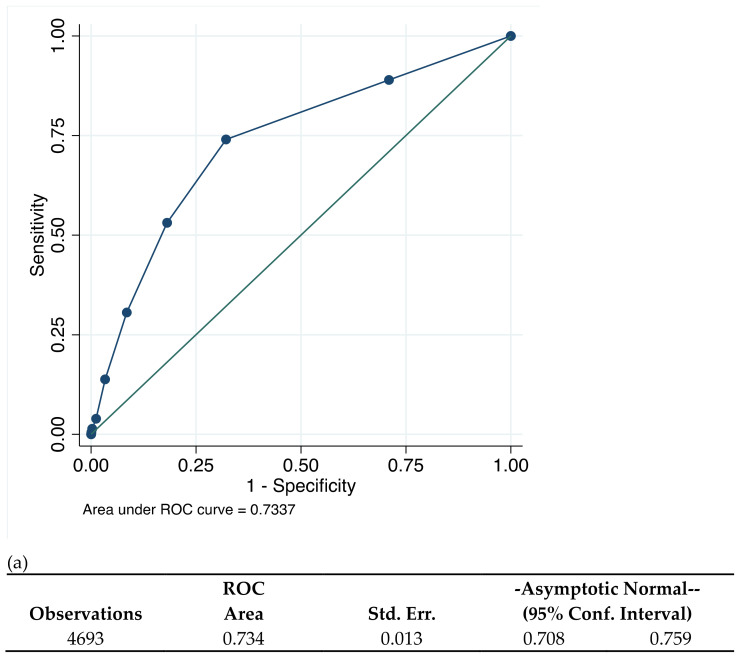

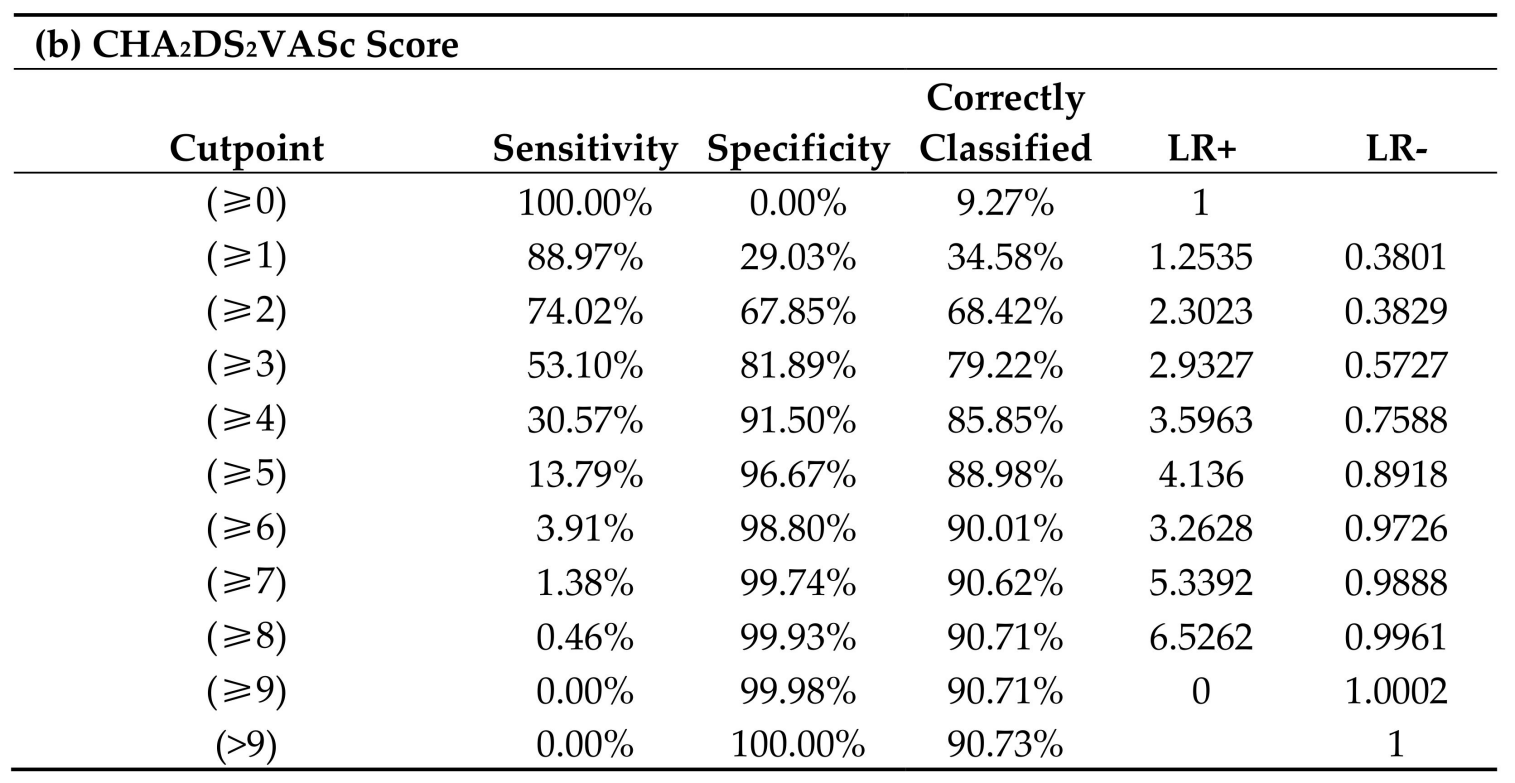
(**a**) CHA_2_DS_2_VASc score and prediction of new-onset AF and (**b**) correctly classified individual by CHA_2_DS_2_VASc scores.

Figure 2 illustrates that the CHA_2_DS_2_VASc scores had an intermediate predictive value for incident AF amongst patients with a history of dental periapical abscess, with AUC 0.73 (95% CI, 0.71–0.76). This AUC correctly predicted 79.2% of patients for new-onset AF with CHA_2_DS_2_VASc score ≥ 3 with a specificity of patients at 53.1% and sensitivity 81.9%. CHA_2_DS_2_VASc scores ≥ 2 were correctly classified at 68.4%, with sensitivity of 74.0% and specificity of 67.9%.

## 4. Discussion

In this study to investigate the occurrence of new-onset AF in patients who were hospitalised because of a dental periapical abscess, our principal findings are as follows: (i) dental periapical abscess acted as an independent predictor for new onset AF; (ii) the CHA_2_DS_2_VASc score in patients with acute dental periapical abscess has significant predictive value for incident AF; and (iii) other associations of new-onset AF during follow-up in patients with dental periapical abscess were older age, male sex, hypertension, aortic regurgitation, dilated cardiomyopathy, previous pacemaker or defibrillator and inflammatory disease.

To our knowledge, this is the first study to investigate patients with acute dental infections and hospitalisation, as a population-based cohort study for new-onset AF, although as discussed previously, a recent systematic review by Hassan et al. has summarised that there is a need for more research in this area [27]. This is important given that hospitalisations because of dental abscesses are not uncommon—for example, a retrospective analysis in the United States found that over 61,000 patients had been hospitalised for this reason over an 8 year period, and a total of 66 people had died [39].

Although outwith the main empirical focus of our paper, it is useful to consider biological plausibility and the evidence for possible mechanisms linking acute dental infections to AF. Dental periapical abscess represents an immune response from infected pulp tissue mainly due to Streptococcus, Prevotella and Fusobacterium species [40]. The presence of different Gram-positive and Gram-negative oral bacteria induce conflict between microbial pathogens and host immune system, resulting in severe pain and destruction of the surrounding dental tissues [41,42]. The presence of different Gram-positive and Gram-negative oral bacteria in the bloodstream can induce cytokines interleukin-1 (IL-1 β), tumour necrosis factor (TNF-α) and metalloproteinases (MMPs), leading to a dysregulated acute response, and in some instances leading to sepsis, a common life-threatening complication when the body’s immune system responds to infection and injures its own tissues and organs [43,44,45]. It is also known that proinflammatory markers (IL-1, TNF-α) and MMPs associated with acute dental infections and cellular remodelling are expressed during occurrence of AF [46,47,48]. The mechanisms that are expressed during dental periapical abscess may be consequential to the heart, even if the infection is resolved. Other articles have shown that some types of severe infection, such as sepsis, are associated with new-onset AF, although it is not clear in this study how many patients with hospitalised dental periapical abscess have also suffered with sepsis, therefore more research is needed in this area to understand the impact [49,50].

The CHA_2_DS_2_VASc score is a validated clinical tool used to predict the risk of stroke and thromboembolism for patients with confirmed diagnosis of AF [51]. Given that the CHA_2_DS_2_VASc is a cluster of common cardiovascular risk factors, the score also has been reported to predict adverse events for patients without AF, such as mortality, stroke, cardiovascular events, and subsequent diagnosis of AF [37,52,53]. Prior studies that have investigated oral diseases and AF have never previously evaluated the CHA_2_DS_2_VASc score as a prediction tool for new-onset AF. Our results suggest that CHA_2_DS_2_VASc score may have modest predictive value for predicting AF, as in the general population without oral sepsis. Nonetheless, the CHA_2_DS_2_VASc score was not proposed to predict AF, but to risk stratify for stroke, which may suggest a weakness for the AUC score and design.

### 4.1. Clinical Implications

With an ageing population demographic predicted to involve 25% being over 60 by 2050, more of whom are likely to retain their teeth into old age; and with AF being more prevalent with increased age, implications of links between poor oral health and CVD and AF are of concern to the dental profession and the health system in general [54,55]. Moreover, since the COVID-19 pandemic has led to suspension of routine dental services in many parts of the world since restoring teeth involves aerosol generating procedures, increased numbers of people are attending services with oral and dental infections [56]. Interventions through improving oral health or signposting and screening for AF and CVD may help improve patient care, prevent severe cardiovascular complication, and reduce health care expenditure across the system. Finally, investigating different biomarkers for both acute and chronic oral diseases and their relationship with AF may serve as a unique model for understanding different inflammatory pathways and their relationship with AF and AF-related complications.

### 4.2. Limitations

We investigated dental infection using a hospital database, and although this enables us to monitor large amounts of data, a major limitation is its retrospective design, and it does not imply cause or effect. As in previous studies, our study defined diagnosis of new-onset AF during hospitalisation, rather than using detailed investigations or outpatient appointments, meaning it could underestimate the true incidence of AF. Unadjusted incidence rate of AF was lower in patients with dental periapical abscess, but these patients were markedly younger. We thus used multivariable analysis to account for differences in age and prevalent comorbidities associated with AF for the study population. Although we found dental abscess to be an independent risk factor using multivariable analysis, there may have been some confounders missing from the dataset. Nonetheless, our data accounted for more potential confounders (such as smoking, obesity and alcohol related diagnosis) which have been a limitation for other prior studies investigating oral diseases and AF [22,23,24].

Another possible limitation is that even though we accounted for selection-bias using a nationwide database with the French population, different codes may be more common in different countries and that the data presented may not be generalisable. A cross sectional study in England found dental problems accounted for 0.7% of all attendees in emergency departments and “dental unspecified”, “dental abscess” and “toothache” and were the most common codes used [30]. Currie et al. [30] went on to explain that the true number of patients attending with acute dental periapical abscess may have been underrepresented due to non-dental healthcare staff using codes “dental unspecified” and general abscess (rather than dental). It was not possible to check the categorisation of “periapical abscess” with dental imaging to confirm diagnosis, but we attempted to rectify this problem by excluding other codes used apart from “dental periapical abscess”, which we felt healthcare staff would not have coded unless confident of the diagnosis for the patient. We only included patients that were hospitalised, meaning they were likely to be managed under healthcare staff with specialty knowledge of diagnosing and treating the patient. Dental periapical abscess was included as a primary and secondary diagnosis because even as an additional finding, it would still mean the dental periapical abscess was also a problem if a hospital needed to investigate and diagnose it clinically.

## 5. Conclusions

Our results from this population-based cohort study demonstrate an increased risk of new onset AF for individuals hospitalised with dental periapical abscess. Careful follow up of patients with severe, acute dental infections is needed for incident AF, as well as investigations of possible mechanisms linking these conditions.

## Figures and Tables

**Table 1:** Baseline characteristics of patients seen in French hospitals in 2013 at least 5 years of follow-up (mean follow-up 4.8 ± 1.7 years, median 5.5, IQR 5.1–5.8 years).

	No Periapical Abscess	Periapical Abscess	*p*	Total
	(n = 3,051,598)	(n = 4693)		(n = 3,056,291)
Age, years	57.3 ± 21.4	51.7 ± 19.6	<0.0001	57.2 ± 21.4
Gender (male)	1,399,638 (45.9)	2484 (52.9)	<0.0001	1,402,122 (45.9)
Hypertension	808,382 (26.5)	1345 (28.7)	0.001	809,727 (26.5)
Diabetes mellitus	382,586 (12.5)	848 (18.1)	<0.0001	383,434 (12.5)
Heart failure	18,981 (18.9)	94 (20.9)	0.26	19,075 (18.9)
History of pulmonary edema	14,666 (0.5)	30 (0.6)	0.12	14,696 (0.5)
Valve disease	65,436 (2.1)	188 (4.0)	<0.0001	65,624 (2.1)
Aortic stenosis	28,003 (0.9)	83 (1.8)	<0.0001	28,086 (0.9)
Aortic regurgitation	12,796 (0.4)	35 (0.7)	0.001	12,831 (0.4)
Mitral regurgitation	23,458 (0.8)	68 (1.4)	<0.0001	23,526 (0.8)
Previous endocarditis	2417 (0.1)	42 (0.9)	<0.0001	2459 (0.1)
Dilated cardiomyopathy	41,195 (1.3)	107 (2.3)	<0.0001	41,302 (1.4)
Coronary artery disease	260,194 (8.5)	519 (11.1)	<0.0001	260,713 (8.5)
Previous myocardial infarction	43,172 (1.4)	78 (1.7)	0.15	43,250 (1.4)
Previous PCI	71,028 (2.3)	130 (2.8)	0.04	71,158 (2.3)
Previous CABG	6768 (0.2)	21 (0.4)	0.001	6789 (0.2)
Vascular disease	218,697 (7.2)	479 (10.2)	<0.0001	219,176 (7.2)
Previous pacemaker or ICD	53,716 (1.8)	98 (2.1)	0.09	53,814 (1.8)
Ischemic stroke	42,238 (1.4)	76 (1.6)	0.17	42,314 (1.4)
Intracranial bleeding	26,915 (0.9)	51 (1.1)	0.13	26,966 (0.9)
Smoker	207,727 (6.8)	892 (19.0)	<0.0001	208,619 (6.8)
Dyslipidemia	357,903 (11.7)	687 (14.6)	<0.0001	358,590 (11.7)
Obesity	299,091 (9.8)	579 (12.3)	<0.0001	299,670 (9.8)
Alcohol related diagnoses	167,392 (5.5)	703 (15.0)	<0.0001	168,095 (5.5)
Chronic kidney disease	83,601 (2.7)	176 (3.8)	<0.0001	83,777 (2.7)
Lung disease	266,460 (8.7)	670 (14.3)	<0.0001	267,130 (8.7)
Sleep apnoea syndrome	108,409 (3.6)	184 (3.9)	0.17	108,593 (3.6)
COPD	139,929 (4.6)	375 (8.0)	<0.0001	140,304 (4.6)
Liver disease	99,391 (3.3)	398 (8.5)	<0.0001	99,789 (3.3)
Gastroesophageal reflux	102,057 (3.3)	149 (3.2)	0.52	102,206 (3.3)
Thyroid diseases	139,843 (4.6)	267 (5.7)	0.0003	140,110 (4.6)
Inflammatory disease	152,875 (5.0)	426 (9.1)	<0.0001	153,301 (5.0)
Anaemia	217,291 (7.1)	604 (12.9)	<0.0001	217,895 (7.1)
Previous cancer	443,622 (14.5)	734 (15.6)	0.03	444,356 (14.5)
Poor nutrition	98,432 (3.2)	339 (7.2)	<0.0001	98,771 (3.2)
Cognitive impairment	84,244 (2.8)	163 (3.5)	0.003	84,407 (2.8)
Illicit drug use	13,101 (0.4)	135 (2.9)	<0.0001	13,236 (0.4)
CHA_2_DS_2_VASc score	1.6 ± 1.3	1.5 ± 1.4	<0.0001	1.6 ± 1.3
Death during follow-up	745,315 (24.4)	1234 (26.3)	0.003	746,549 (24.4)
Cardiovascular death	130,554 (4.3)	176 (3.8)	0.07	130,730 (4.3)
Atrial fibrillation during follow-up	326,644 (10.7)	435 (9.3)	0.001	327,079 (10.7)

Values are n (%) or mean ± SD. CABG = coronary artery bypass graft; COPD = chronic obstructive pulmonary disease; PCI = percutaneous coronary intervention; SD = standard deviation.

**Table 2:** CHA_2_DS_2_VASc score at baseline in patients seen in French hospitals in 2013 at least 5 years of follow-up (mean follow-up 4.8 ± 1.7 years, median 5.5, IQR 5.1–5.8 years).

	No Periapical Abscess			Periapical Abscess		
Variables	(n = 3,051,598)			(n = 4693)		
Age, years	57.3 ± 21.4			51.7 ± 19.6		
CHA_2_DS_2_VASc score	1.6 ± 1.3			1.5 ± 1.4		
CHA_2_DS_2_VASc score, n (%)						
Score = 0	626,912	20.54	%	1284	27.36	%
Score = 1	1,175,581	38.52	%	1718	36.61	%
Score = 2	546,836	17.92	%	689	14.68	%
Score = 3	426,115	13.96	%	507	10.8	%
Score = 4	172,369	5.65	%	293	6.24	%
Score = 5	73,417	2.41	%	134	2.86	%
Score = 6	23,678	0.78	%	51	1.09	%
Score = 7	5683	0.19	%	12	0.26	%
Score = 8	875	0.03	%	4	0.09	%
Score = 9	132	0	%	1	0.02	%

**Table 3:** Predictors of new-onset atrial fibrillation during follow-up in the whole population of patients seen in French hospitals in 2013 with at least 5 years of follow-up (mean follow-up 4.8 ± 1.7 years, median 5.5, IQR 5.1–5.8 years).

	Univariate Analysis		Multivariable Analysis	
	HR, 95%CI	*p*	HR, 95%CI	*p*
Age, years	1.077 (1.076–1.077)	<0.0001	1.076 (1.075–1.076)	<0.0001
Gender (male)	1.640 (1.629–1.651)	<0.0001	1.498 (1.487–1.509)	<0.0001
Hypertension	2.849 (2.829–2.869)	<0.0001	1.114 (1.105–1.123)	<0.0001
Diabetes mellitus	1.951 (1.935–1.968)	<0.0001	1.106 (1.096–1.116)	<0.0001
Heart failure	3.893 (3.857–3.930)	<0.0001	1.434 (1.416–1.452)	<0.0001
History of pulmonary edema	2.978 (2.862–3.099)	<0.0001	1.155 (1.108–1.203)	<0.0001
Aortic stenosis	4.862 (4.763–4.963)	<0.0001	1.406 (1.376–1.436)	<0.0001
Aortic regurgitation	3.343 (3.237–3.453)	<0.0001	1.131 (1.094–1.170)	<0.0001
Mitral regurgitation	3.580 (3.496–3.665)	<0.0001	1.298 (1.266–1.332)	<0.0001
Previous endocarditis	2.970 (2.735–3.225)	<0.0001	1.343 (1.236–1.459)	<0.0001
Dilated cardiomyopathy	3.321 (3.260–3.383)	<0.0001	1.236 (1.211–1.262)	<0.0001
Coronary artery disease	2.805 (2.780–2.830)	<0.0001	1.110 (1.097–1.124)	<0.0001
Previous myocardial infarction	2.128 (2.082–2.176)	<0.0001	0.903 (0.880–0.926)	<0.0001
Previous PCI	2.043 (2.010–2.077)	<0.0001	0.888 (0.871–0.906)	<0.0001
Vascular disease	2.521 (2.496–2.546)	<0.0001	1.091 (1.078–1.104)	<0.0001
Sinus node disease	3.903 (3.781–4.029)	<0.0001	1.200 (1.161–1.240)	<0.0001
Previous pacemaker or ICD	4.423 (4.357–4.490)	<0.0001	1.319 (1.298–1.340)	<0.0001
Ischemic stroke	2.289 (2.239–2.340)	<0.0001	1.140 (1.114–1.165)	<0.0001
Intracranial bleeding	1.374 (1.322–1.427)	<0.0001	0.885 (0.852–0.920)	<0.0001
Smoker	0.903 (0.891–0.917)	<0.0001	1.052 (1.036–1.069)	<0.0001
Dyslipidemia	1.835 (1.819–1.851)	<0.0001	0.879 (0.871–0.888)	<0.0001
Obesity	1.385 (1.371–1.399)	<0.0001	1.270 (1.256–1.285)	<0.0001
Alcohol related diagnoses	0.991 (0.975–1.006)	0.24	1.289 (1.267–1.312)	<0.0001
Chronic kidney disease	2.516 (2.480–2.554)	<0.0001	1.262 (1.243–1.282)	<0.0001
Lung disease	1.885 (1.866–1.905)	<0.0001	1.108 (1.090–1.126)	<0.0001
Sleep apnoea syndrome	1.579 (1.556–1.603)	<0.0001	1.115 (1.098–1.133)	<0.0001
COPD	2.316 (2.287–2.344)	<0.0001	1.111 (1.089–1.133)	<0.0001
Liver disease	1.141 (1.119–1.164)	<0.0001	1.082 (1.059–1.105)	<0.0001
Gastroesophageal reflux	0.756 (0.740–0.772)	<0.0001	0.804 (0.787–0.821)	<0.0001
Thyroid diseases	1.316 (1.297–1.336)	<0.0001	0.985 (0.970–0.999)	0.04
Inflammatory disease	1.036 (1.020–1.052)	<0.0001	0.978 (0.964–0.994)	0.005
Anaemia	1.772 (1.751–1.793)	<0.0001	1.082 (1.068–1.095)	<0.0001
Previous cancer	1.602 (1.587–1.617)	<0.0001	1.055 (1.045–1.065)	<0.0001
Poor nutrition	1.775 (1.744–1.807)	<0.0001	0.951 (0.934–0.969)	<0.0001
Cognitive impairment	2.368 (2.326–2.410)	<0.0001	0.821 (0.807–0.836)	<0.0001
Illicit drug use	0.288 (0.263–0.317)	<0.0001	0.940 (0.855–1.032)	0.19
Periapical abscess	0.855 (0.778–0.939)	0.001	1.107 (1.008–1.216)	0.03

COPD = chronic obstructive pulmonary disease; PCI = percutaneous coronary intervention.

**Table 4:** Predictors of new-onset atrial fibrillation during follow-up in patients with periapical abscess seen in French hospitals in 2013 with at least 5 years of follow-up (mean follow-up 4.8 ± 1.7 years, median 5.5, IQR 5.1–5.8 years).

	Univariate Analysis		Multivariable Analysis	
	HR, 95%CI	*p*	HR, 95%CI	*p*
Age, years	1.074 (1.067–1.081)	<0.0001	1.068 (1.061–1.075)	<0.0001
Gender (male)	1.475 (1.216–1.789)	<0.0001	1.580 (1.324–1.885)	<0.0001
Hypertension	3.877 (3.202–4.695)	<0.0001	1.234 (1.020–1.493)	0.03
Diabetes mellitus	1.798 (1.456–2.220)	<0.0001	1.173 (0.954–1.442)	0.13
Heart failure	4.014 (3.217–5.008)	<0.0001	1.200 (0.898–1.603)	0.22
History of pulmonary edema	1.803 (0.579–5.613)	0.31	0.483 (0.171–1.365)	0.17
Aortic stenosis	6.962 (4.796–10.106)	<0.0001	1.130 (0.690–1.850)	0.63
Aortic regurgitation	2.246 (1.062–4.751)	0.03	2.038 (1.108–3.749)	0.02
Mitral regurgitation	4.721 (3.072–7.255)	<0.0001	1.080 (0.604–1.930)	0.80
Previous endocarditis	4.097 (2.307–7.275)	<0.0001	2.839 (0.955–8.443)	0.06
Dilated cardiomyopathy	3.324 (2.216–4.987)	<0.0001	1.973 (1.273–3.058)	0.002
Coronary artery disease	3.493 (2.825–4.319)	<0.0001	1.158 (0.886–1.512)	0.28
Previous myocardial infarction	2.767 (1.745–4.390)	<0.0001	0.762 (0.434–1.337)	0.34
Previous PCI	2.428 (1.643–3.587)	<0.0001	0.814 (0.519–1.275)	0.37
Vascular disease	3.241 (2.602–4.036)	<0.0001	1.036 (0.793–1.353)	0.80
Sinus node disease	5.828 (3.165–10.729)	<0.0001	1.628 (0.875–3.032)	0.12
Previous pacemaker or ICD	4.542 (3.074–6.711)	<0.0001	1.758 (1.242–2.488)	0.001
Ischemic stroke	3.096 (1.849–5.186)	<0.0001	1.233 (0.796–1.909)	0.35
Intracranial bleeding	1.045 (0.391–2.799)	0.93	0.773 (0.319–1.873)	0.57
Smoker	0.680 (0.517–0.895)	0.006	1.059 (0.782–1.434)	0.71
Dyslipidemia	2.153 (1.735–2.671)	<0.0001	0.757 (0.601–0.953)	0.02
Obesity	1.803 (1.428–2.277)	<0.0001	1.080 (0.846–1.380)	0.54
Alcohol related diagnoses	0.731 (0.540–0.990)	0.04	1.181 (0.857–1.629)	0.31
Chronic kidney disease	2.735 (1.950–3.835)	<0.0001	1.229 (0.888–1.701)	0.21
Lung disease	1.453 (1.134–1.861)	0.003	1.161 (0.815–1.653)	0.41
Sleep apnoea syndrome	2.141 (1.514–3.026)	<0.0001	0.943 (0.673–1.322)	0.74
COPD	2.120 (1.601–2.808)	<0.0001	0.948 (0.612–1.469)	0.81
Liver disease	1.068 (0.752–1.516)	0.71	1.134 (0.778–1.653)	0.51
Gastroesophageal reflux	0.788 (0.443–1.400)	0.42	1.060 (0.672–1.671)	0.80
Thyroid diseases	1.361 (0.954–1.941)	0.09	1.119 (0.788–1.590)	0.53
Inflammatory disease	0.956 (0.693–1.320)	0.79	1.416 (1.079–1.859)	0.01
Anaemia	1.587 (1.217–2.068)	0.001	0.881 (0.660–1.176)	0.39
Previous cancer	1.738 (1.349–2.239)	<0.0001	0.999 (0.801–1.246)	0.99
Poor nutrition	1.329 (0.926–1.907)	0.12	0.755 (0.467–1.221)	0.25
Cognitive impairment	1.565 (0.963–2.545)	0.07	0.678 (0.439–1.047)	0.08
Illicit drug use	0.447 (0.200–1.001)	0.05	0.449 (0.110–1.831)	0.26

COPD = chronic obstructive pulmonary disease; PCI = percutaneous coronary intervention.

**Table 5:** Incidence (per 100 person-years) of new-onset atrial fibrillation with different CHA_2_DS_2_VASc scores in patients with periapical abscess seen in French hospitals in 2013 with at least 5 years of follow-up (mean follow-up 4.8 ± 1.7 years, median 5.5, IQR 5.1–5.8 years).

	Number of Patients	Person-Years	Number of New-Onset AF	Incidence Rate, %/year (95%CI)
CHA_2_DS_2_-VASc Score				
Score = 0	1284	6413.57	48	0.748 (0.564–0.993)
Score = 1	1718	8643.09	65	0.752 (0.590–0.959)
Score = 2	689	2927.79	91	3.108 (2.531–3.817)
Score = 3	507	1930.53	98	5.076 (4.165–6.188)
Score = 4	293	1109.12	73	6.582 (5.233–8.279)
Score = 5	134	405.27	43	10.610 (7.869–14.306)
Score = 6	51	172.17	11	6.389 (3.538–11.537)
Score = 7	12	40.15	4	9.964 (3.740–26.547)
Score = 8	4	8.27	2	24.172 (6.045–96.649)
Score = 9	1	0.16	0	0.000
Total	4693	21,650.12	435	2.009 (1.829–2.207)

**Table 6:** Hazard ratio of new-onset atrial fibrillation in patients with acute dental periapical abscess using different CHA_2_DS_2_VASc scores (in comparison to the patients with score of 0).

	Number of Patients	Number of New-Onset AF	Hazard Ratio (95% CI)	*p* Value
CHA_2_DS_2_-VASc Score				
Score = 0 (reference)	1257	48	1	-
Score = 1	1708	65	0.983 (0.677–1.428)	0.93
Score = 2	690	91	4.056 (2.859–5.755)	<0.0001
Score = 3	531	98	6.353 (4.494–8.983)	<0.0001
Score = 4	371	73	8.616 (5.983–12.408)	<0.0001
Score = 5	190	43	13.315 (8.808–20.129)	<0.0001
Score = 6	84	11	8.289 (4.300–15.979)	<0.0001
Score = 7	20	4	6.205 (2.063–18.657)	0.001
Score = 8	5	2	34.189 (8.300–140.835)	<0.0001
Score = 9	1	0	-	-

## Data Availability

The French Data Protection Authority have granted access to the PMSI data between 2008 and 2015, for the purpose of assessing the prevalence and incidence of cardiovascular diseases and their complications to the University of Tours in France.
